# Differences in Seed Weight, Amino Acid, Fatty Acid, Oil, and Squalene Content in γ-Irradiation-Developed and Commercial Amaranth Varieties (*Amaranthus* spp.)

**DOI:** 10.3390/plants9111412

**Published:** 2020-10-22

**Authors:** Monika Szabóová, Michal Záhorský, Ján Gažo, Jeroen Geuens, Ann Vermoesen, Els D’Hondt, Andrea Hricová

**Affiliations:** 1 Plant Science and Biodiversity Center, Institute of Plant Genetics and Biotechnology, Slovak Academy of Sciences, 95007 Nitra, Slovakia; m.szaboova@savba.sk (M.S.); michal.zahorsky@gmail.com (M.Z.); 2 Department of Genetics and Plant Breeding, Faculty of Agrobiology and Food Resources, Slovak University of Agriculture, 94901 Nitra, Slovakia; jan.gazo@uniag.sk; 3 The Centre of Expertise on Sustainable Chemistry, KdG University of Applied Sciences and Arts, 2018 Antwerpen, Belgium; jeroen.geuens@kdg.be (J.G.); ann.vermoesen@kdg.be (A.V.); els.dhondt@kdg.be (E.D.)

**Keywords:** amaranth, mutation, nutrition, amino acids, fatty acids, squalene

## Abstract

Grain amaranth is known as an alternative crop with exclusive nutritional value and health benefits. The purpose of this study was to investigate the effect of gamma irradiation on quantitative and qualitative amaranth seed traits, including 1000-seed weight, amino acids, fatty acids content, oil, and squalene yield. Two Slovak mutant varieties “Pribina” (*A. cruentus*) and “Zobor” (*A.* *hypochondriacus x A. hybridus*) were evaluated and compared to nonirradiated controls Ficha (*A. cruentus* L.) and K-433 (*A. hypochondriacus x A. hybridus*) and commercial varieties, Aztec (*A. cruentus* L.), Plainsman and Koniz (*A. hypochondriacus x A. hybridus*). Mutant varieties, “Pribina” and “Zobor”, showed superior 1000-seed weight performance compared to all investigated amaranth samples. The change in quantitative seed trait was accompanied by significantly higher oil and squalene content compared to commercial varieties. Moreover, significantly higher content of essential linoleic acid was detected in mutant variety “Zobor”. The present findings suggest that seeds of irradiation-derived varieties have high nutritional potential and can be used as a supplementary crop in the human diet.

## 1. Introduction

Amaranth is a multipurpose crop that can be used as food and animal feed, as well as an ornamental plant [1]. Amaranth grain has higher nutritional value compared to traditional cereals and several legumes with high protein and well-balanced essential amino acid level, especially lysine and methionine [2,3,4]. In contrast to typical grains, amaranth proteins are mainly formed of albumins and globulins with little or no content of prolamine proteins. Prolamins are the major storage proteins in cereals and are involved in imparting certain intolerances, such as gluten-sensitivity [5]. The main aspect of protein value in amaranth grain is its essential amino acid composition (EAA). The lysine content is twice that of wheat and three times higher compared to maize. Hovewer, amino acid and protein quantity in amaranth depends on the genotype and year of production [3,6]. Usually, lysine and valine are the limiting amino acids in most cereals [7]. Bressani et al. [8] reported threonine as the limiting amino acid in amaranth grain. However, others [9,10] reported that leucine was the limiting amino acid followed by valine and threonine. Despite low content of leucine, isoleucine, and valine in amaranth grain, these are not considered to be a problem in the human diet since they can be found in most common grains. Therefore, amaranth is suitable for blending with cereals in bread making [11].

The lipid content in amaranth seed is up to three times higher compared to buckwheat and common cereals [12]. The fatty acid (FA) composition is an important characteristic of fat and oil and determines their nutritional, technological, and stability properties [6,13,14]. Amaranth oil consists of approximately 77% unsaturated FA with high level of linoleic acid, which is essential for human nutrition [15]. Antioxidative and related properties of amaranth oil are often linked with squalene, which is a type of unsaponifiable lipid and acts as a biosynthetic precursor to all steroids in plants and animals. Squalene, the main component of skin surface polyunsaturated lipids, shows some advantages for the skin as an emollient and antioxidant and for hydration and its antitumor activities [16,17]. Several studies confirmed the health benefits of squalene in nutritional, medicinal, and pharmaceutical aspects, and squalene is even considered a potential chemopreventive and chemotherapeutic agent [17,18,19]. Shark liver oil contains large quantities of squalene and is considered its richest source (2300–8400 mg/100 g oil). Besides shark liver oil, several plants were reported to be a source of squalene including as rice, wheat germ, and grapes (<1.0%), followed by palm oil, soybean, maize, and macadamia, reviewed in [20]. The highest yield was recorded for olive (150–747 mg/100 g oil) and amaranth (6000–8000 mg/100 g oil), which are considered to be the most valuable source of this important bioactive molecule. Out of these species, only olives are used for the commercial extraction of squalene, although the highest content is reported for amaranth oil [20].

Amaranth is also considered a good source of insoluble fiber and has high vitamin and mineral content, such as riboflavin, niacin, ascorbic acid, calcium, and magnesium [5,21]. Amaranth seeds contain a high level of calcium which has special relevance for people suffering from celiac disease due to the well known prevalence of osteopenia and osteoporosis among celiacs [5].

One of the tools for improving seed traits of important crops is mutation breeding, which has been successfully utilized for variety development and generating polygenic variability [22,23]. Our previous research focused on the improvement of quality and quantity traits in amaranth through γ-radiation [24,25,26,27,28,29].

Hence, we aimed at the comparison of selected quantitative and qualitative seed traits in two gamma-ray derived Slovak varieties “Pribina” (*Amaranthus cruentus*) and “Zobor” (*Amaranhtus hypochondriacus* × *Amaranthus hybridus*), in their nonirradiated control genotype Ficha (*A. cruentus*) and hybrid K-433 (*Amaranhtus hypochondriacus* × *Amaranthus hybridus*), and in the preferred commercial varieties Aztec (*Amaranthus cruentus*), Plainsman and Koniz (*Amaranhtus hypochondriacus* × *Amaranthus hybridus*). We evaluated 1000-seed weight as an important yield parameter and determined the content of important seed nutritional properties, such as amino acids, total oil, fatty acids, and squalene.

## 2. Results and Discussion

### 2.1. Evaluation of 1000-seed Weight

The variation in quantitative traits is conditioned by both genetic and environmental factors [30,31]. One of the important scales in seed quality that influences germination, seed vigor, seedling establishment, and yield is 1000-seed weight [32]. Herein, the 1000-seed weight was investigated during three consecutive growing seasons (2016–2018) for seven grain amaranth genotypes and varieties (Supplementary Table S1).

**Figure 1:**
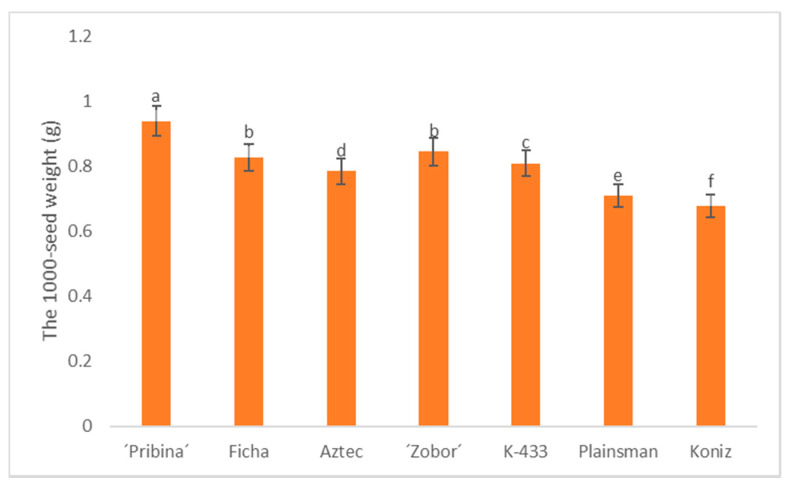
Comparison of 1000-seed weight over a three year period (2016–2018) in amaranth samples. Results are the means (± SE; standard error) for three growing seasons of three independent biological replicates per each variety per year. Different letters on bars indicate significant differences by the Tukey test (*p* ≤ 0.05).

Notably, γ-radiation-derived varieties “Pribina” and “Zobor”, followed by nonirradiated control Ficha, produced the highest 1000-seed weight among tested samples (Table 1, Figure 1). In variety “Pribina”, seed weight was within the range of 0.90–0.97 g, while variety “Zobor” showed comparable weight of thousand seeds (0.83–0.86 g) to genotype Ficha (0.77–0.87 g).

In addition, the obtained data demonstrated significant differences between newly bred varieties “Pribina” and “Zobor” in comparison to commercially available grain varieties, Aztec (0.73–0.82 g), Plainsman (0.68–0.73 g), and Koniz (0.68–0.71 g).

Among the tested amaranth samples, the lowest 1000-seed weight was found in commercial varieties Koniz and Plainsman, especially when harcested in 2018 (0.68 g for both). Similar results of 1000-seed weight in similar conditions, i.e., Central European conditions, have been previously reported [33,34,35].

Gimplinger et al. [35] analyzed grain quality and grain yield in *Amaranthus cruentus* and *Amaranthus hypochondriacus*. Authors reported that 1000-seed weight ranged from 0.62 to 0.93 g and was influenced by genotype. Other authors [36] reported that especially pale seeded amaranth genotypes had a 1000-seed weight up to 0.96 g.

Rivelli et al. [37] investigated grain yield and components in 11 different amaranth genotypes, with the 1000-seed weight varying among tested *Amaranthus cruentus* accessions from 0.33 to 0.76 g. Our results showed up to 67% higher 1000-seed weights compared to this report.

Moreover, we observed an effect of the growing season on this tested yield parameter (Table 1). The latter effect can be caused due to climate conditions during vegetation. From our previous results, it is evident that the effect of locality on seed weight was not present but a genotype × year interaction was found [38]. Hovewer, new varieties “Pribina” and “Zobor” demonstrated sTable 1000-seed weight performance over several cropping seasons (Figure 1).

As a result of mutagenesis treatment, demonstrated by significant differences (*p* ≤ 0.05) between mutant varieties “Pribina” and “Zobor” and their nonirradiated counterparts Ficha and K-433, a long-term increase in 1000-seed weight of mutant varieties was observed. Considering that high yield is influenced by seed parameters, such as 1000-seed weight, it is one of the crucial breeding targets for the improvement of yield. As both mutant varieties had higher 1000-seed weight, they are very important for the market.

### 2.2. Amino Acid Analysis

**Figure 2:**
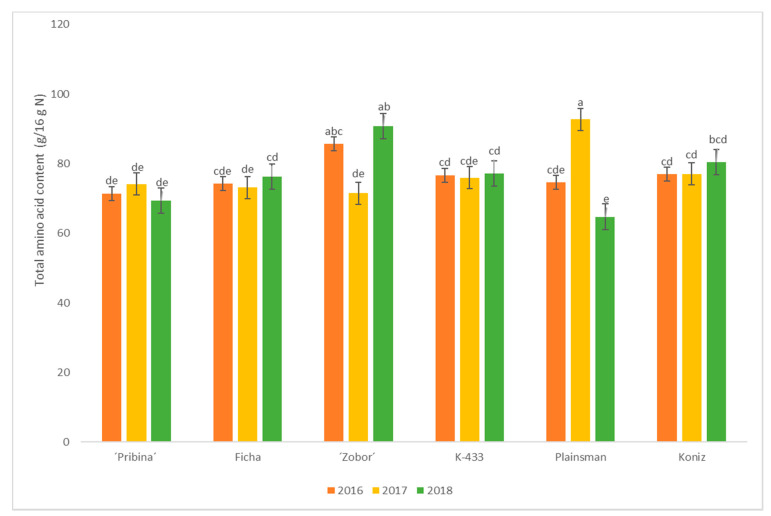
Total amino acid content (g/16 g N) in amaranth seed samples for three cropping seasons (2016–2018). Results are the means (± SE; standard error) for three seasons of three independent biological replicates per each variety per year. Different letters on bars indicate significant differences by the Tukey test (*p* ≤ 0.05).

The determination of total amino acid (AA) content in seeds of mutant varieties “Pribina” and “Zobor”, controls Ficha and hybrid K-433, and commercial varieties Plainsman and Koniz during three consecutive growing seasons (2016–2018) were performed. Figure 2 shows the total AA content in analyzed amaranth seed samples.

A significantly higher (*p* ≤ 0.05) content of summed AA was found in seeds of the commercial variety Plainsman (92.76 g/16 g N), followed by mutant variety “Zobor” (90.75 g/16 g N) in 2017 and 2018, respectively (Figure 2). For comparison, Dodok et al. [39] reported a total AA content of 84.62 g/16 g N in *Amaranthus hypochondriacus* whole flour.

The most abundand AA in analyzed amaranths were glutamic acid (14.85 ± 0.61–17.56 ± 1.87 g/16 g N) and aspartic acid (6.55 ± 0.137.53 ± 1.20 g/16 g N) from NEAA and arginine (6.16 ± 0.34–7.66 ± 1.29 g/16 g N) from EAA (Table 2). These results are similar to other reports for *Amaranthus* [2,6,40,41]. The highest values of these AA were found in grains of variety “Zobor”.

The advantage of amaranth seeds over cereal grains is a relatively high protein content. The quality protein is determined by balanced AA composition, especially with a significant amount of EAA, like lysine and suplhur-containing methionine. Generally, the AA profile of amaranth seeds is more similar to *Leguminosae* than to cereals, except for sulphur-containing AA present in higher amounts in amaranth than in *Leguminosae* [41,42]. Palombini et al. [40] presented that *Amaranthus cruentus* grains have a similar AA composition to *Chenopodium quinoa* but the concentration of essential leucine, lysine, and phenylalanine is higher. Hovewer, the AA content depends on the amaranth species and cultivar [4,6].

The lysine content of amaranth samples analyzed herein was, on average 4.33 g/16 g N (Table 2), with the highest lysine content found in variety “Zobor”(4.70 ± 0.55 g/16 g N). No significant differences were found among the tested amaranth samples. Dodok et al. [39] detected 5.95 g/16 g N of lysine in *Amaranthus hypochondriacus* whole flour. Shukla et al. [43] reported a lysine content between 0.66 g/16 g N and 11.12 g/16 g N in 218 amaranth accessions. The authors reported promising accessions with a lysine content > 7.50 g/16 g N. All evaluated amaranths had comparable methionine levels, ranging from 1.83 ± 0.01 g/16 g N in variety “Zobor” to 2.23 ± 0.46 g/16 g N in hybrid K-433 (Table 2). Our data are similar to those published by other authors [6,44,45]. Mattila et al. [46] analyzed the nutritional value of commercial protein-rich plant sources such as faba bean, lupin, flaxseed, and buckwheat. The total EAA amount ranged from 25.80 g/16 g N in oil hemp peel to 41.50 g/16 g N in pearled quinoa. EAA values detected in our study were comparable to that of whole lupin reported by Mattila et al. [46].

As reported by Kugbe et al. [47], irradiation can induce alterations in the genome that can positively modify the chemical composition and nutritional quality of food crops, giving the breeders an opportunity to select induced mutants with desirable traits. Mehlo et al. [48] demonstrated the change in storage proteins in sorghum after gamma radiation treatment, accompanied by high content of several essential amino acids in the endosperm.

**Figure 3:**
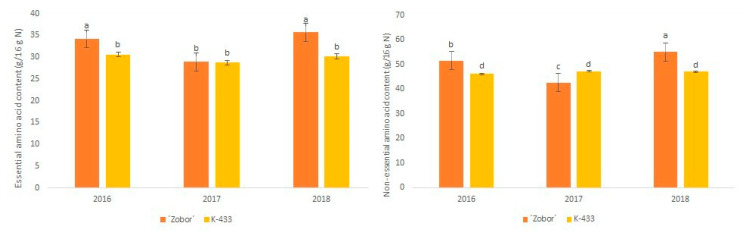
Effect of the growing season on contents of the total essential and nonessential amino acids in mutant variety “Zobor” and control K-433. Different letters on bars indicate significant differences by the Tukey test (*p* ≤ 0.05).

Mutant variety “Zobor” showed a significantly higher (*p* ≤ 0.05) content of essential isoleucine (2.09 ± 0.04 g/16 g N) and valine (2.30 ± 0.05 g/16 g N), and nonessential tyrosine (2.75 ± 0.30 g/16 g N) ompared to nonirradiated control K-433 (1.43 ± 0.16 g/16 g N; 1.63 ± 0.14 g/16 g N; 2.11 ± 0.27 g/16 g N, respectively) during the monitored period (Table 2). Moreover, statistical analysis confirmed significantly higher (*p* ≤ 0.05) content of both EAA and NEAA in mutant variety “Zobor” over the hybrid K-433 in 2016 and 2018 (Figure 3).

In our study, the significant differences found between mutant variety “Zobor” and its control counterpart K-433 were an effect of the growing season rather than irradiation treatment. We presume that inferior performance of “Zobor” in 2017 can be affected by meteorogical conditions, especially the dry period at the beginning of this particular growing season (Supplementary Figure S1)

Additionally, several authors [49,50,51] observed phenotypic and genotypic correlations among several quality and quantity traits, indicating the relative importance of heritability and environmental influences. Sarker et al. [49] reported high heritability values for nutritional traits, such as protein content (83.33%) and Fe content (99.96%), suggesting strong genetic control for these traits. Differences in AA content were reported to be a result of interactions among genetic makeup, environmental factors, and cultural practices [52]. The growing season had a highly significant effect on seed protein and AA concentration, as reported by Mlakar et al. [53].

In the present investigation, the commercially preferred variety Plainsman showed the highest content of EAA (37.60 g/ 16 g N) and NEAA (55.15 g/16 g N) in 2017. Hovewer, the lowest content of summed EAA (24.91 g/ 16 g N), as well as NEAA (39.80 g/16 g N), was also detected in this variety in season 2018, indicating the instability of Plainsman in this analyzed trait. Although variety “Zobor” exhibited relatively unstable performance in 2017, except for methionine and cysteine, the content of all tested AA in “Zobor” seeds was the higher than all other analyzed samples (Table 2). Thus, variety “Zobor” can be considered as a promising new pseudocereal germplasm for the human diet.

**Figure 4:**
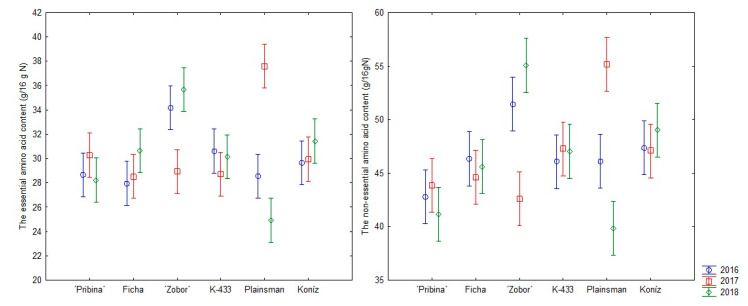
Comparison of consistency in content of essential and and nonessential amino acids over three growing seasons in amaranth samples. Individual columns represent 95% confidence intervals.

The most consistent AA performance was observed for hybrids K-433 and Koniz (Figure 4). Interestingly, K-433 and Koniz have the same genetic background (*Amaranthus hypochondriacus x Amaranthus hybridus*) as Plainsman and “Zobor” showing some degree of instability. Shukla’s findings [43] indicate the existence of a notable diversity for nutritional factors in amaranth germplasm. Sarker et al. [49] suggest the necessity to intercross promising accessions in order to create a larger variation for the effective selection in segregating generations in order to develop nutritionally superior genotypes.

### 2.3. Total Oil and Squalene Content

Amaranth seeds generally have a higher lipid content than common cereals, but it is less in comparison to oilseeds.

The crude oil content in the analyzed amaranth seeds (Table 3) ranged from 4.57 ± 0.29% (Plainsman) to 5.55 ± 0.39% (’Zobor’). Similar results were found by other authors [5,12,54]. A significantly higher (*p* ≤ 0.05) oil content was detected in mutant varieties “Zobor” (5.55 ± 0.39%) and “Pribina” (5.42 ± 0.32%), followed by genotype Ficha (5.41 ± 0.56%), and hybrid K-433 (5.41 ± 0.23%) over commercially known Aztec (4.86 ± 0.51%), Koniz (4.82 ± 0.40%), and Plainsman (4.57 ± 0.29%).

He and Corke [55] reported an average of 4.66% oil in *Amaranthus hybridus*, 4.58% in *Amaranthus hypochondriacus,* and 3.21% oil in *Amaranthus cruentus* seeds, whilst Zhang et al. [14] determined a higher oil content in vegetable *Amaranthus dubius* and *Amaranthus tricolor* seeds (7.77 and 8.91%, respectively). According to the reports, oil content was influenced by amaranth species, cultivar, agrotechnical practices, and growing location. This is also supported by the findings of other authors [54,56]. They determined oil contents in *Amaranthus cruentus* and *Amaranthus hypochondriacus* from 6.39 to 8.20%, whereas in another study oil levels ranged from 1.09 to 5.00% in *Amaranthus cruentus* and from 3.03 to 5.97% in *Amaranthus hypochondriacus* [13].

Squalene (SQ) is one of the unsaturated hydrocarbons of triterpene that is synthesized in plants, animals, fungi, and bacteria as a precursor for the synthesis of hormones, vitamins, and sterols [57]. Squalene is probably the most important element of the unsaponifiable oil fraction in amaranth showing many benefits on human health [58]. One of the main features of SQ is its antioxidant, antibacterial, and antifungal quality [59]. Its content in some commercial oils is relatively low, e.g., 0.2% in coconut [19], 0.5% in palm oil, 0.7% in flaxseed, 1% in olive, and 1.5% in avocado. Among plant sources, olive is the only commercially used crop to extract SQ, despite that the amaranth seed is reported to have the highest content of this beneficial substance [20,60].

Mutant variety “Pribina” showed the highest SQ value (6.94 ± 0.70%), while the lowest content was detected in variety Aztec (3.85 ± 1.20%; Table 3). Different extraction methods are used to define the SQ content in particular natural extracts. In our study, the SQ content was determined in the oil. Subsequently, by measuring the oil content of the seeds, the SQ amount in the seed was calculated (Table 3).

**Figure 5:**
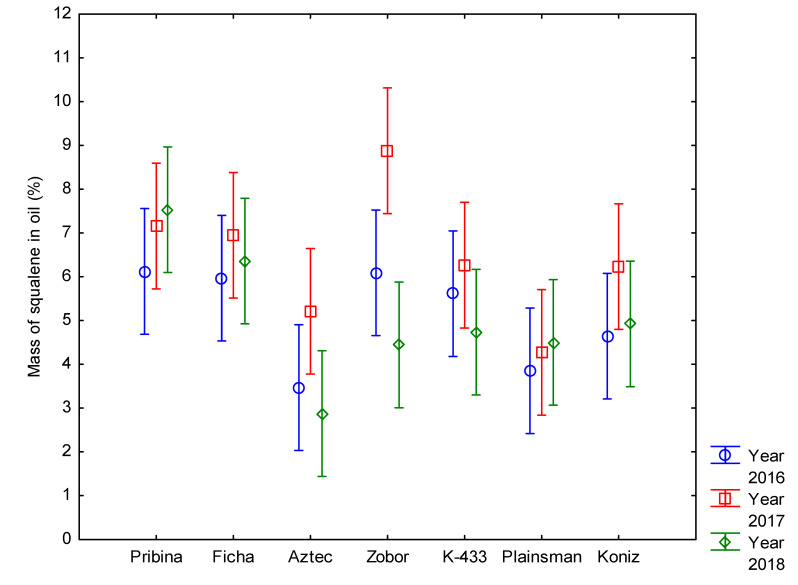
Comparison of consistency in content of squalene over three cropping seasons in amaranth oil. Individual columns represent 95% confidence intervals.

Among seven amaranth samples tested during three growing seasons, the *Amaranthus cruentus* mutant variety “Pribina” and its control genotype Ficha were found to be the most stable in SQ content over a tested period (Figure 5). Lozano-Grande et al. [20] reported variations in SQ yield, depending on the species, harvest season, postharvest conditions, and the extraction method when testing potential plant sources, such as *Olea europea*, *Amaranthus* spp., *Glycine max*, and *Zea mays*. Regardless of the different factors or used extraction method, amaranth has the highest content of SQ that can be up to approximately 11% [61].

We can conclude that the seeds of the mutant varieties “Pribina” and “Zobor”, and genotype Ficha, can be considered an exclusively rich plant source of SQ in comparison to other investigations [13,55,62,63]. Furthermore, both mutagenesis-derived varieties had significantly higher (*p* ≤ 0.05) oil and SQ yield compared to commercial varieties, Plainsman, Aztec, and Koniz.

### 2.4. Fatty Acid Composition

Oil extracted from amaranth grain (up to 9%, according to Gamel et al. [63]) is attractive due to its high unsaturated FA level and several bioactive constituents [14]. In general, the composition of FA in oil determines its nutritional properties but also oil stability [43].

The FA composition of the analyzed amaranth samples tested in our study during three seasons (Table 4) was in accordance with previously published results [6,13,54,61,62]. As expected, unsaturated FA were represented at the highest level (74.71 ± 1.05%–5.46 ± 0.26%). Linoleic acid (39.71 ± 1.83%–46.06 ± 4.43%) and oleic acid (26.25 ± 3.43%–33.13 ± 2.95%) were the most abundant FA, followed by palmitic acid (19.67 ± 0.48%–20.14 ± 0.25%). Stearic acid and linolenic acid were present in lower amounts (3.36 ± 0.29%–3.67 ± 0.08%; 0.69±0.04%–0.75 ± 0.06%, respectively). The remaining FA occured in very low amounts. Statistical analyses showed significant differences (*p* ≤ 0.05) only for myristic, oleic, and linoleic acid content in the analyzed amaranth seeds (Table 4).

The average content of linoleic acid was around 42%, which was similar to findings reported by Hlinková et al. [56] who analyzed 10 amaranth genotypes with an average linoleic acid content of 40.90% in Plainsman and 42.60% in Koniz. The highest content of linoleic acid was detected in mutant variety “Zobor” (46.06%) and was about 11% higher compared to its nonirradiated control K-433.

The results showed an average content of oleic acid of 30.86% (Table 4). Hlinková et al. [54] reported a yield of oleic acid between 20.30 and 22.85% for Koniz and Plainsman, respectively. Other studies have shown variation between 23.80 and 31.72% in *Amaranthus cruentus* [63] and 14.90 and 30.20% in *Amaranthus hypochondriacus* [54]. Moreover, the relationship between oleic acid/linoleic acid ratios and SQ amounts was reported [64].

Mutant variety “Zobor” exhibited a significantly lower content of oleic acid, which may be caused by the negative correlation between linoleic and both oleic and palmitic acids [54,55]. In the study of Berganza et al. [62], grain variety GUA-17 (*Amaranthus cruentus*) showed the lowest content of oleic acid (23.80%) but the highest content of linoleic acid (40.70%).

The content of saturated palmitic acid varied between 19.67% (Pribina) and 20.14% (’Zobor’), which is in accordance with most published data [12,62,65]. However, others [54] detected an average value of palmitic acid in *Amaranthus hypochondriacus* (31.50%) and in *Amaranthus* cruentus (31.30%). The content of another saturated FA, stearic acid, ranges according to different species and different authors reported yields from 0.1% in *Amaranhus asplundii* to 4.1% in *Amaranthus dubius* [14,55,56]. The average content of stearic acid detected in our study was 3.51%.

The values of α-linolenic acid in the analyzed amaranth seeds ranged from 0.69 to 0.75%. Similar data were previously obtained by Jahaniaval et al. [61] and León-Camacho et al. [66]. They detected 0.65 and 0.93% α-linolenic acid in the seeds of *Amaranthus cruentus* and *Amaranthus hypochondriacus*, respectively.

The saturated FA comprised on average 24.03% of the total FA and the unsaturated FA 75.00%, leading to an average saturated/unsaturated ratio of 0.32. Similar values were determined by other authors [14,55,56].

Our results confirmed previous reports, suggesting amaranth oil as a good source of unsaturated FA with high levels of linoleic acid suitable for human nutrition [15].

## 3. Materials and Methods

### 3.1. Plant Material and 1000-seed Weight

Two grain amaranth species were evaluated in the present study: *Amaranthus cruentus* L. (genotype Ficha, and varieties Aztek and “Pribina”) and *Amaranthus hypochondriacus x Amaranthus hybridus* (accession K-433, and varieties Koniz, Plainsman, and “Zobor”). Slovak mutant varieties “Pribina” and “Zobor” were bred at the home institute—Institute of Plant Genetics and Biotechnology by mutation breeding [25,38]. Briefly, mutant lines were generated by radiation mutagenesis from the initial control varieties (Ficha, K-433) using a gamma radiation dose of 175 Gy [24] in the Joint FAO/IAEA Programme Agency’s laboratories in Seibersdorf, Austria. After the evaluation of 12 generations of previously induced mutants, two new varieties had a significant increase in 1000-seed weight. Seed samples of nonmutated amaranth varieties were obtained from the collection of GenBank from the Crop Research Institute Praha-Ruzyňe, Czech Republic.

Amaranth plants were cultivated in three consecutive years (2016–2018) at the locality of Nitra (290 m above sea level; 48° 18′ 53.442” N, 18° 5′ 16.75” E), with a mean annual precipitation of 600 mm and a mean annual temperature of 9.5 °C. The soil type at the experimental field in Nitra is a Haplic Luvisol with pH of 7.4. Weather conditions (average temperature and rainfall) in the vegetation season of all three years are shown in Supplementary Figure S1.

Seeds were manually sowed in May and plants were harvested in September in each year. The seeds were hand-threshed from dried panicles. During the growing season, no fertilizers or sprays were applied.

The weight of 1000 seeds was calculated as an average value of 10 independent measurements for each sample.

### 3.2. Amino Acid Analysis

Essential amino acids: lysine (Lys), histidine (His), arginine (Arg), threonine (Thr), valine (Val), isoleucyne (Ile), leucine (Leu), methionine (Met), phenylalanine (Phe) and nonessential amino acids: aspartic acid (Asp), glutamic acid (Glu), serine (Ser), proline (Pro), glysine (Gly), alanine (Ala), tyrosine (Tyr), and cysteine (Cys) were analyzed in amaranth seeds from all amaranth samples (in duplicate) except variety Aztec. The content of amino acids after acid hydrolysis (6M HCl) and sulphur amino acids after oxidation hydrolysis (33% H_2_O_2_ and 85 % HCOOH. 1:9) was determined in an automatic amino acid analyzer AAA 400 (Ingos, Praha, Czech Republic) and results were analyzed using CHROMuLAN software. Seeds and leaves were homogenized for both procedures, and a 0.5 g sample was used. Hydrolysis was carried out at 110 °C while refluxing under a stream of nitrogen for 23 h.

### 3.3. Oil Analysis

#### 3.3.1. Oil Content and Squalene Content

For the purpose of oil and squalene content in the amaranth seeds harvested during period 2016–2018, all analyses were performed in triplicate.

The optimized procedure for amaranth sample pretreatment involved drying the seeds in a vacuum oven at 90 °C for 2 h, then adding 50 g of dry seeds on top of milling balls, and milling at 400 rpm for 3 min. All milling steps were performed in a Pulverisette 6 planetary mono mill (Fritsch). Extractions were performed in an ASE350 apparatus (Dionex). Twelve grams of seed powder were mixed with 1.1 g of Celite powder and added to a 22 mL extraction cell. The extraction solvent was 100% n-hexane, the extractuion emperature was 70 °C, the static time was 10 min, and 3 extraction cycles were performed. After extraction, the solvent was evaporated using a rotavapor apparatus (Büchi). The temperature of the water bath was set at 60 °C and the vacuum was set at 500 mbar. After evaporation, the flasks were left to stand open overnight for any residual solvent to be removed, after which the mass of oil was recorded. The amount of squalene in the oil was determined by gas chromatography (Trace 1300, Thermo Scientific, Louvain-la-Neuve, Belgium) using a capillary MXT-5 column (30 m × 0.53 mm, film thickness 0.25 µm, Restek) equipped with a SSL injector (inlet temperature 350 °C) and FID detector (380 °C, constant flow, hydrogen 35 mL/min, air 350 mL/min, nitrogen 40 mL/min) under a temperature gradient (50 °C for 1 min, ramp to 180 °C at 10 °C/min; ramp to 230 °C at 3 °C/min; ramp to 380 °C at 15 °C/min; 380 °C for 10 min). A constant helium flow of 7.0 mL/min was applied.

Samples for GC analysis were prepared by mixing 0.1 g of a 10 m% oil solution in n-heptane with 0.04 g of a 1 m% tetradecane solution in n-heptane (internal standard solution) and diluting to 1 g with n-heptane. To perform quantitative analysis of squalene a calibration was performed using a squalene standard (Acros Organics). Analysis results were evaluated using Chromeleon 6 software.

#### 3.3.2. Fatty Acid Composition

The gas chromatography (GC-6890-N, Agilent Technologies, Santa Clara, USA) equipped with capillary column DB-23 (60 m × 0.25 mm, film thickness 0.25 µm, Agilent Technologies, Santa Clara, CA, USA) and FID detector (250 °C; constant flow, hydrogen 40 mL/min, air 450 mL/min.) was used for amaranth oil metyl esters fatty acids (FAME) profile analysis [67]. Temperature gradient was as follows: 150 °C for 3 min., ramp to 175 °C at 7 °C/min; 175 °C for 5 min; ramp to 195 °C at 5 °C/min; ramp to 225 °C at 4.5 °C/min; 225 °C for 0.5 min; ramp to 215 °C at 10 °C/min; 215 °C for 7 min; ramp to 240 °C at 10 °C/min; 240 °C held for 7 min. Hydrogen was used as a carrier gas (flow 2.5 mL/min; velocity 57 cm/s; pressure 220 kPa). Split ratio was 1/20 (inlet temperature 230 °C; hydrogen flow of 51 mL/min for 2 min; then hydrogen flow of 20 mL/min; pressure 220 kPa). Standards of a C4–C24 FAME mixture (Supelco, Bellefonte, PA, USA) were applied in order to identify FAME peaks. The evaluation was carried out by the ChemStation 10.1 software. Analysis of fatty acid composition was established in a duplicate of each sample in each year.

### 3.4. Experiment Design and Statistical Analysis

The experimental field design was a block in a split plot arrangement with four replications. For each experimental variant, the plot size was 2.0 m × 1.5 m (3 m^2^). Isolation between each amaranth variety was secured by a corn plot (1 m wide).

The 1000-seed weight was calculated as an average value of 10 independent measurements for each variety per each year. Results of of AA, crude oil, and SQ content are the means for three growing seasons of three independent biological replicates per each variety. Results of FA content are the means for three growing seasons of two independent biological replicates per each variety.

The data were subjected to statistical analysis using Statistica 10 software (StatSoft Inc., Tulsa, OK, USA). Analysis of variance (ANOVA), Tukey HSD multiple comparison test, and Fisher LSD test were employed to identify significant differences among the different processes at *p* ≤ 0.05.

## 4. Conclusions

The mutant varieties analyzed in this report exhibited stable and consistently superior performance of 1000-seed weight over nonirradiated controls and commercial varieties during a three year period. The content of all tested amino acids in mutant variety “Zobor” seed was the highest from all analyzed amaranth samples, except for methionine and cysteine. In addition, “Zobor” seeds had a significantly higher content of essential linoleic acid. Furthermore, both mutagenesis-derived varieties “Pribina” and “Zobor” had significantly higher oil and squalene yield compared to the three tested commercial varieties: Aztec, Plainsman, and Koniz. Thus, these two Slovak varieties can be used by farmers and producers as promising new germplasms generated by induced mutagenesis.

## Figures and Tables

**Table 1:** The 1000-seed weight of investigated amaranth samples tested during three consecutive growing seasons.

Year	*A. cruentus* L.			*A. hypochondriacus x A. hybridus*				$\bar{x}$ ± SE
	‘Pribina’	Ficha	Aztec	‘Zobor’	K-433	Plainsman	Koniz	
**2016**	0.97 ^a^	0.87 ^b^	0.73 ^e^	0.83 ^c^	0.82 ^d^	0.72 ^f^	0.71 ^f^	0.81 ± 0.09
**2017**	0.95 ^a^	0.84 ^c^	0.78 ^d^	0.86 ^b^	0.84 ^c^	0.73 ^e^	0.69 ^f^	0.81 ± 0.09
**2018**	0.90 ^b^	0.77 ^e^	0.82 ^d^	0.84 ^c^	0.77 ^e^	0.68 ^f^	0.68 ^f^	0.78 ± 0.08

Different letters (a–f) indicate significant differences by the Tukey test (*p* ≤ 0.05). Results are the means. (± SE; standard error) for three growing seasons of 10 independent biological replicates per each variety per year.

**Table 2:** Contents of essential and nonessential amino acids (g/16 g N) in amaranth seeds in three year period (2016–2018).

Amino Acid	*A. cruentus* L.		*A. hypochondriacus x A. hybridus*			
	’Pribina’	Ficha	’Zobor’	K-433	Plainsman	Koniz
**Essential Amino Acid**						
**Arginine (Arg)**	6.16 ± 0.34 ^b^	6.23 ± 0.14 ^ab^	7.66 ± 1.29 ^a^	7.13 ± 0.01 ^ab^	6.63 ± 1.55 ^ab^	7.04 ± 0.45 ^ab^
**Histidine (His)**	2.37 ± 0.09 ^c^	2.43 ± 0.47 ^bc^	2.91 ± 0.45 ^ab^	3,02 ± 0.17 ^a^	2.61 ± 0.25 ^abc^	2.91 ± 0.05 ^ab^
**Isoleucine (Ile)**	2.15 ± 0.20 ^a^	2.03 ± 0.33 ^ab^	2.09 ± 0.04 ^a^	1.43 ± 0.16 ^b^	1.96 ± 0.76 ^ab^	1.61 ± 0.07 ^ab^
**Leucine (Leu)**	3.94 ± 0.42 ^a^	3.97 ± 0.42 ^a^	4.71 ± 0.65 ^a^	4.32 ± 0.19 ^a^	4.36 ± 0.71 ^a^	4.47 ± 0.03 ^a^
**Methionine (Met)**	2.00 ± 0.24 ^a^	2.18 ± 0.40 ^a^	1.83 ± 0.01 ^a^	2.23 ± 0.46 ^a^	2.13 ± 0.32 ^a^	2.13 ± 0.04 ^a^
**Phenylalanine (Phe)**	2.74 ± 0.34 ^a^	2.79 ± 0.64 ^a^	3.35 ± 0.38 ^a^	2.85 ± 0.21 ^a^	3.21 ±0.64 ^a^	3.20 ± 0.12 ^a^
**Threonine (Thr)**	3.06 ± 0.19 ^a^	2.99 ± 0.42 ^a^	3.41 ± 0.32 ^a^	2.92 ± 0.09 ^a^	3.10 ± 0.75 ^a^	2.98 ± 0.10 ^a^
**Lysine (Lys)**	4.23 ± 0.31 ^a^	4.22 ± 0.13 ^a^	4.70 ± 0.55 ^a^	4.30 ± 0.07 ^a^	4.26 ± 0.92 ^a^	4.25 ± 0.18 ^a^
**Valine (Val)**	2.40 ± 0.29 ^a^	2.22 ± 0.36 ^abc^	2.30 ± 0.05 ^ab^	1.63 ± 0.14 ^c^	2.09 ± 0.72 ^abc^	1.74 ± 0.09 ^bc^
**Nonessential Amino Acid**						
**Alanine (Ala)**	2.91 ± 0.16 ^b^	3.12 ± 0.08 ^ab^	3.79 ± 0.85 ^a^	3.14 ± 0.27 ^ab^	3.04 ± 0.46 ^b^	3.32 ± 0.14 ^ab^
**Aspartic acid (Asp)**	6.55 ± 0.13 ^a^	6.60 ± 0.08 ^a^	7.53 ± 1.20 ^a^	7.09 ± 0.11 ^a^	6.96 ± 0.94 ^a^	7.17 ± 0.23 ^a^
**Cysteine (Cys)**	1.78 ± 0.13 ^b^	2.13 ± 0.06 ^a^	1.73 ± 0.13 ^b^	2.10 ± 0.04 ^a^	2.01 ± 0.13 ^a^	2.07 ± 0.03 ^a^
**Glutamic acid (Glu)**	14.85 ± 0.61 ^a^	16.25 ± 0.80 ^a^	17.56 ± 1.87 ^a^	16.36 ± 0.15 ^a^	17.17 ± 3.35 ^a^	16.84 ± 0.54 ^a^
**Glycine (Gly)**	5.74 ± 0.13 ^b^	6.14 ± 0.21 ^ab^	6.69 ± 0.94 ^a^	6.78 ±0.30 ^a^	6.22 ± 0.76 ^ab^	6.69 ± 0.10 ^a^
**Pro (Proline)**	3.12 ± 0.22 ^a^	3.42 ± 0.11 ^a^	3.67 ± 0.34 ^a^	3.34 ± 0.02 ^a^	3.10 ± 0.99 ^a^	3.42 ± 0.11 ^a^
**Ser (Serine)**	5.25 ± 0.07 ^ab^	5.14 ± 0.17 ^b^	5.99 ± 0.96 ^a^	5.88 ± 0.03 ^ab^	5.80 ± 0.57 ^ab^	5.84 ± 0.13 ^ab^
**Tyr (Tyrosine)**	2.39 ± 0.07 ^ab^	2.71 ± 0.13 ^a^	2.75 ± 0.30 ^a^	2.11 ± 0.27 ^b^	2.71 ± 0.57 ^a^	2.47 ± 0.13 ^ab^

Different letters (a–c) indicate significant differences (within each row) by the Tukey test (*p* ≤ 0.05). Results are the means (± SE; standard error) for three growing seasons of three independent biological replicates per each variety per year.

**Table 3:** Crude oil and squalene content (%) in amaranth seeds in three year period (2016–2018).

	*A. cruentus* L.			*A. hypochondriacus x A. hybridus*			
	’Pribina’	Ficha	Aztec	’Zobor’	K-433	Plainsman	Koniz
**Oil**	5.42 ± 0.32 ^a^	5.41 ± 0.56 ^a^	4.86 ± 0.51 ^b^	5.55 ± 0.39 ^a^	5.41 ± 0.23 ^a^	4.57 ± 0.29 ^c^	4.82 ± 0.45 ^b^
**Squalene in oil**	6.94 ± 0.70 ^a^	6.42 ± 0.47 ^ab^	3.85 ± 1.20 ^d^	6.47 ± 0.83 ^ab^	5.54 ± 0.67 ^b^	4.21 ± 0.35 ^cd^	5.26 ± 1.08 ^bc^
**Squalene in seeds**	0.38 ± 0.05 ^a^	0.35 ± 0.02 ^ab^	0.19 ± 0.08 ^d^	0.36 ± 0.19 ^ab^	0.30 ± 0.05 ^bc^	0.19 ± 0.01^d^	0.26 ± 0.07 ^cd^

Different letters (a–d) indicate significant differences by LSD test (*p* ≤ 0.05). Results are the means (± SE; standard error) for three growing seasons of three independent biological replicates per each variety per year.

**Table 4:** Content of fatty acids in amaranth seeds (%).

Fatty Acid	*A. cruentus* L.		*A. hypochondriacus x A. hybridus*	
	’Pribina’	Ficha	’Zobor’	K-433
**Myristic acid (C14:0)**	0.19 ± 0.00 ^a^	0.19 ± 0.01 ^a^	0.17 ± 0.02 ^b^	0.19 ± 0.01 ^a^
**Palmitic acid** **(C16:0)**	19.67 ± 0.48 ^a^	19.81 ± 0.31 ^a^	20.14 ± 0.25 ^a^	19.88 ± 0.63 ^a^
**Palmitoleic acid (C16:1 cis)**	0.15 ± 0.02 ^a^	0.15 ± 0.02 ^a^	0.13 ± 0.02 ^a^	0.15 ± 0.02 ^a^
**Stearic acid** **(C18:0)**	3.41 ± 0.30 ^a^	3.60 ± 0.16 ^a^	3.67 ± 0.08 ^a^	3.36 ± 0.29 ^a^
**Oleic acid (C 18:1 cis)**	32.55 ± 1.53 ^a^	33.13 ± 2.95 ^a^	26.25 ± 3.43 ^b^	31.52 ± 1.85 ^a^
**Linoleic acid (C18:2 cis)**	40.49 ± 0.55 ^b^	39.71 ± 1.83 ^b^	46.06 ± 4.43 ^a^	41.41 ± 0.70 ^b^
**α-Linolenic acid (C18:3 cis)**	0.70 ± 0.02 ^a^	0.74 ± 0.04 ^a^	0.75 ± 0.06 ^a^	0.69 ± 0.04 ^a^
**Arachidic acid (C20:0)**	0.50 ± 0.26 ^a^	0.50 ± 0.24 ^a^	0.48 ± 0.25 ^a^	0.49 ± 0.26 ^a^
**Eicosenoic acid (C20:1 cis)**	0.33 ± 0.08 ^a^	0.34 ± 0.09 ^a^	0.26 ± 0.02 ^a^	0.31 ± 0.06 ^a^
**Saturated**	23.77 ± 0.08 ^b^	23.95 ± 0.29 ^b^	24.46 ± 0.08 ^a^	23.93 ± 0.16 ^b^
**Unsaturated**	75.07 ± 0.75 ^a^	75.46 ± 0.26 ^a^	74.74 ± 0.37 ^a^	74.71 ± 1.05 ^a^
**Saturated/Unsaturated**	0.32 ± 0.00 ^b^	0.32 ± 0.00 ^b^	0.33 ± 0.00 ^a^	0.32 ± 0.00 ^b^

Different letters (a–b) indicate significant differences (within each row) by the LSD test (*p* ≤ 0.05). Results are the means (± SE; standard error) for three growing seasons of two independent biological replicates per each variety per year.

## Supplementary Materials

The following are available online at https://www.mdpi.com/2223-7747/9/11/1412/s1. Table S1: The 1000-seed weight of amaranth samples during three year period. Figure S1: Meteorological conditions during the amaranth cropping season from 2016–2018 in locality Nitra.
