# Supplementary material for: Differences in Seed Weight, Amino Acid, Fatty Acid, Oil, and Squalene Content in γ-Irradiation-Developed and Commercial Amaranth Varieties (Amaranthus spp.)
Source: Plants (Basel). 2020 Oct 22;9(11):1412. doi: 10.3390/plants9111412 (PMC7690577; doi:10.3390/plants9111412)
Supplement: Supplementary file 1 [file plants-09-01412-s001.zip › supplementary/plants-969555-supplementary-proofdone.docx]

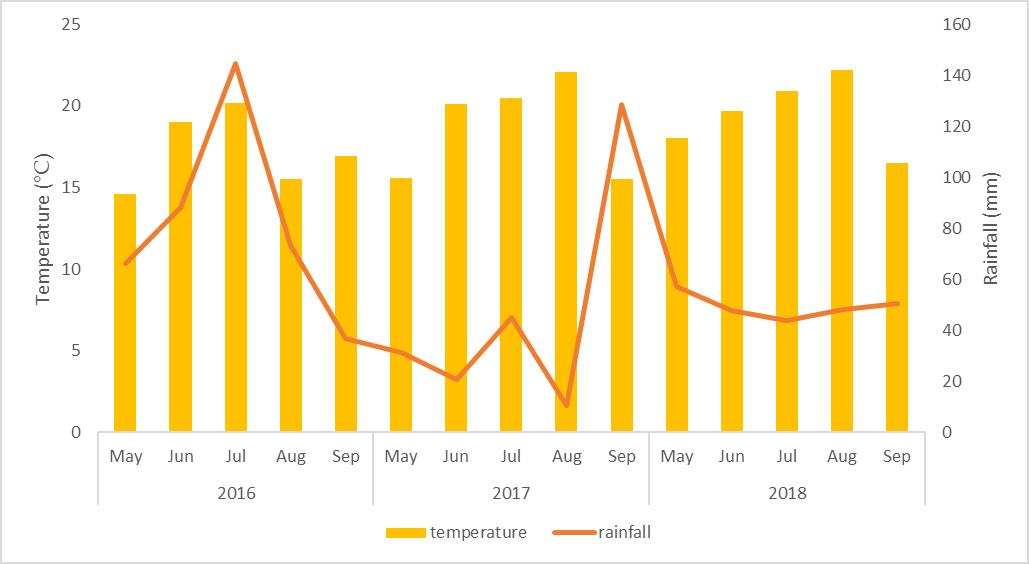


**Supplementary Figure 1.** Meteorological conditions during the amaranth cropping season from 2016–2018 in locality Nitra.
