# Supplementary material for: Differences in Seed Weight, Amino Acid, Fatty Acid, Oil, and Squalene Content in γ-Irradiation-Developed and Commercial Amaranth Varieties (Amaranthus spp.)
Source: Plants (Basel). 2020 Oct 22;9(11):1412. doi: 10.3390/plants9111412 (PMC7690577; doi:10.3390/plants9111412)
Supplement: Supplementary file 1 [file plants-09-01412-s001.zip › supplementary/plants-969555-supplementary.pdf]

Table S1. The 1000-seed weight of amaranth samples during three years period

Year	Variety	1000-seed weight (g)										$\bar{x}$
2016	´Pribina´	0.96	0.97	0.96	0.97	0.97	0.97	0.96	0.97	0.97	0.99	0.97
	Ficha	0.86	0.86	0.87	0.87	0.88	0.86	0.87	0.88	0.87	0.87	0.87
	Aztec	0.78	0.75	0.78	0.76	0.76	0.75	0.74	0.73	0.76	0.77	0.76
	´Zobor´	0.83	0.84	0.84	0.84	0.83	0.82	0.84	0.84	0.83	0.82	0.83
	K-433	0.82	0.80	0.80	0.82	0.83	0.83	0.82	0.82	0.81	0.80	0.82
	Plainsman	0.73	0.70	0.73	0.72	0.72	0.72	0.71	0.73	0.73	0.72	0.72
	Koniz	0.71	0.70	0.71	0.71	0.70	0.71	0.72	0.72	0.71	0.71	0.71
2017	´Pribina´	0.94	0.94	0.95	0.95	0.95	0.94	0.95	0.95	0.95	0.94	0.95
	Ficha	0.85	0.84	0.84	0.84	0.84	0.84	0.84	0.84	0.84	0.84	0.84
	Aztec	0.78	0.79	0.77	0.77	0.78	0.77	0.77	0.78	0.79	0.79	0.78
	´Zobor´	0.87	0.86	0.86	0.86	0.86	0.86	0.86	0.87	0.87	0.86	0.86
	K-433	0.84	0.84	0.83	0.84	0.83	0.84	0.83	0.83	0.84	0.83	0.84
	Plainsman	0.73	0.73	0.74	0.72	0.73	0.73	0.73	0.74	0.73	0.72	0.73
	Koniz	0.65	0.65	0.65	0.63	0.63	0.65	0.64	0.65	0.69	0.64	0.69
2018	´Pribina´	0.91	0.87	0.89	0.91	0.90	0.92	0.91	0.90	0.91	0.90	0.90
	Ficha	0.77	0.77	0.77	0.77	0.78	0.77	0.77	0.77	0.78	0.79	0.77
	Aztec	0.81	0.84	0.83	0.80	0.82	0.82	0.82	0.81	0.80	0.81	0.82
	´Zobor´	0.83	0.85	0.86	0.83	0.84	0.83	0.85	0.83	0.85	0.80	0.84
	K-433	0.79	0.75	0.76	0.76	0.76	0.79	0.77	0.78	0.78	0.78	0.77
	Plainsman	0.67	0.68	0.67	0.68	0.67	0.70	0.69	0.67	0.68	0.67	0.68
	Koniz	0.69	0.68	0.70	0.69	0.70	0.67	0.66	0.62	0.68	0.68	0.68
